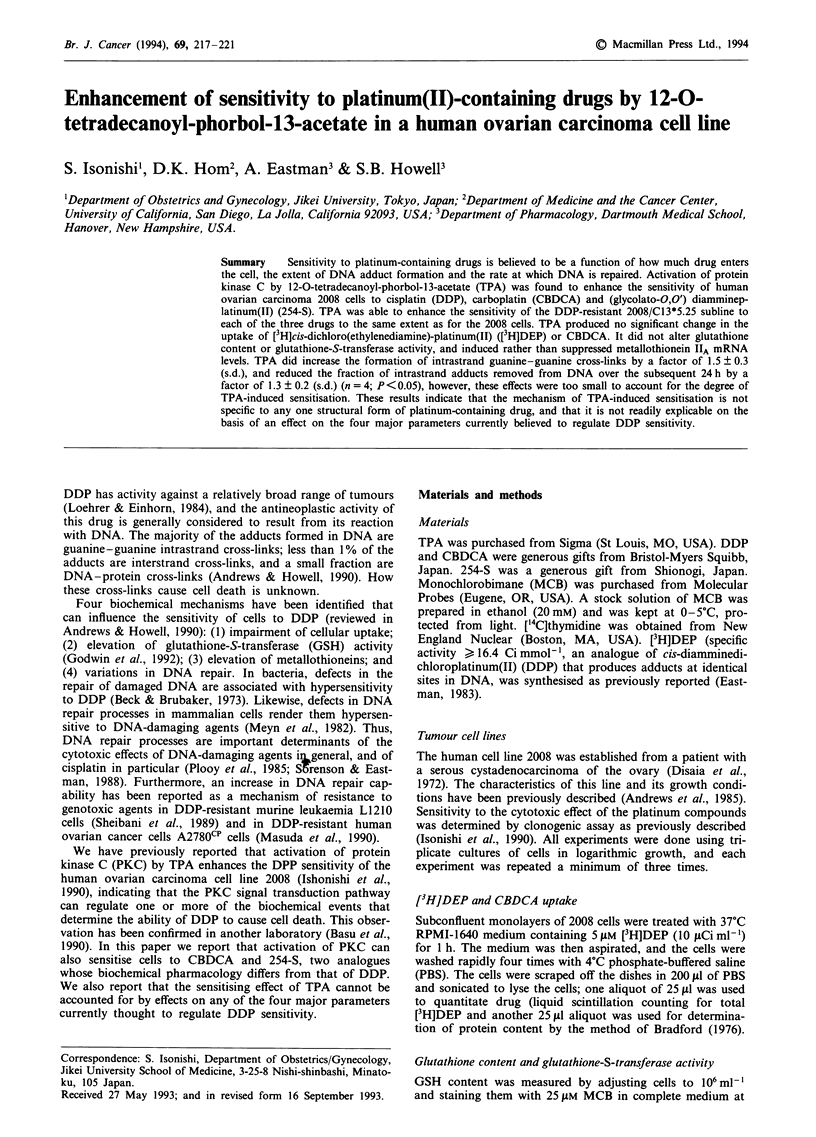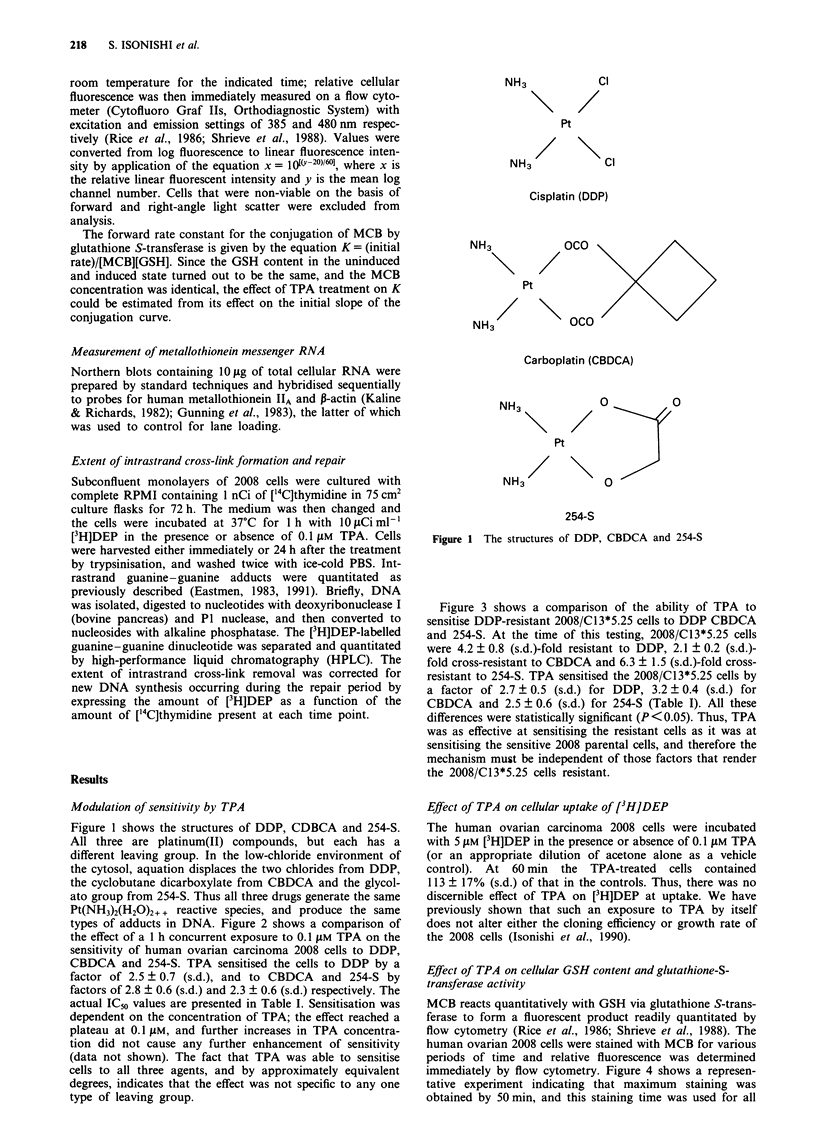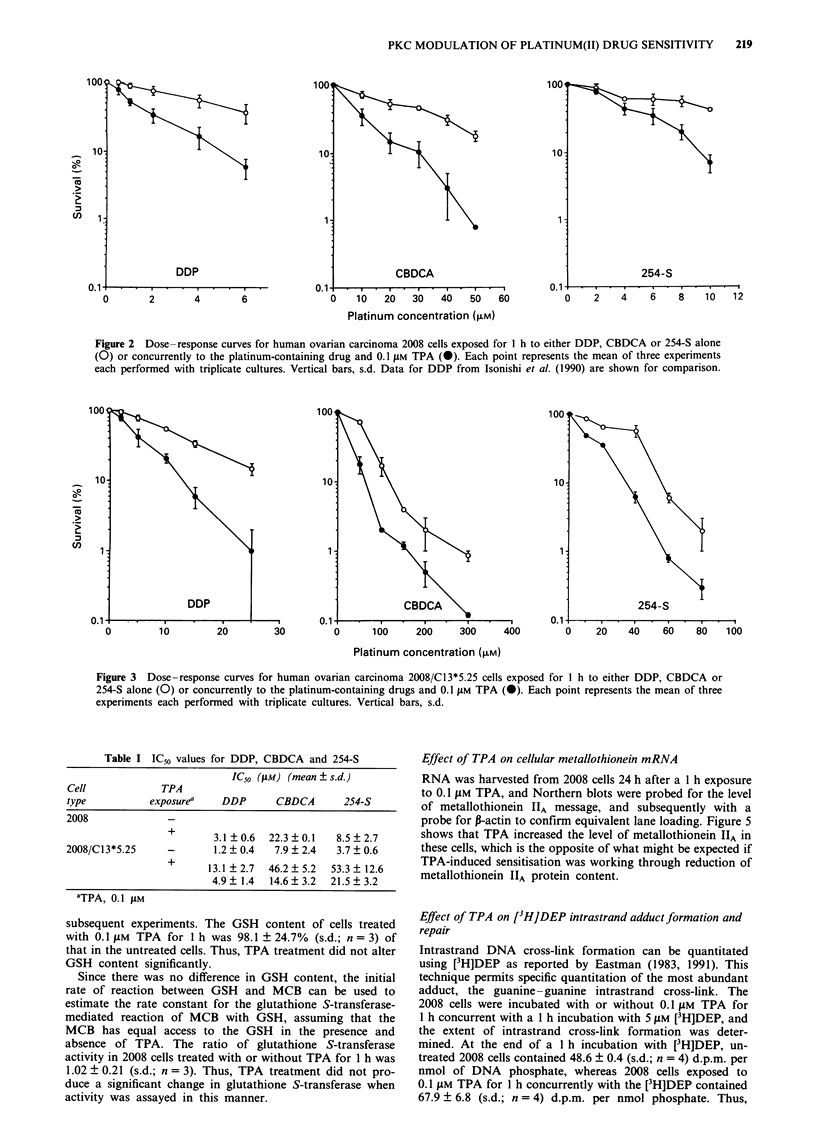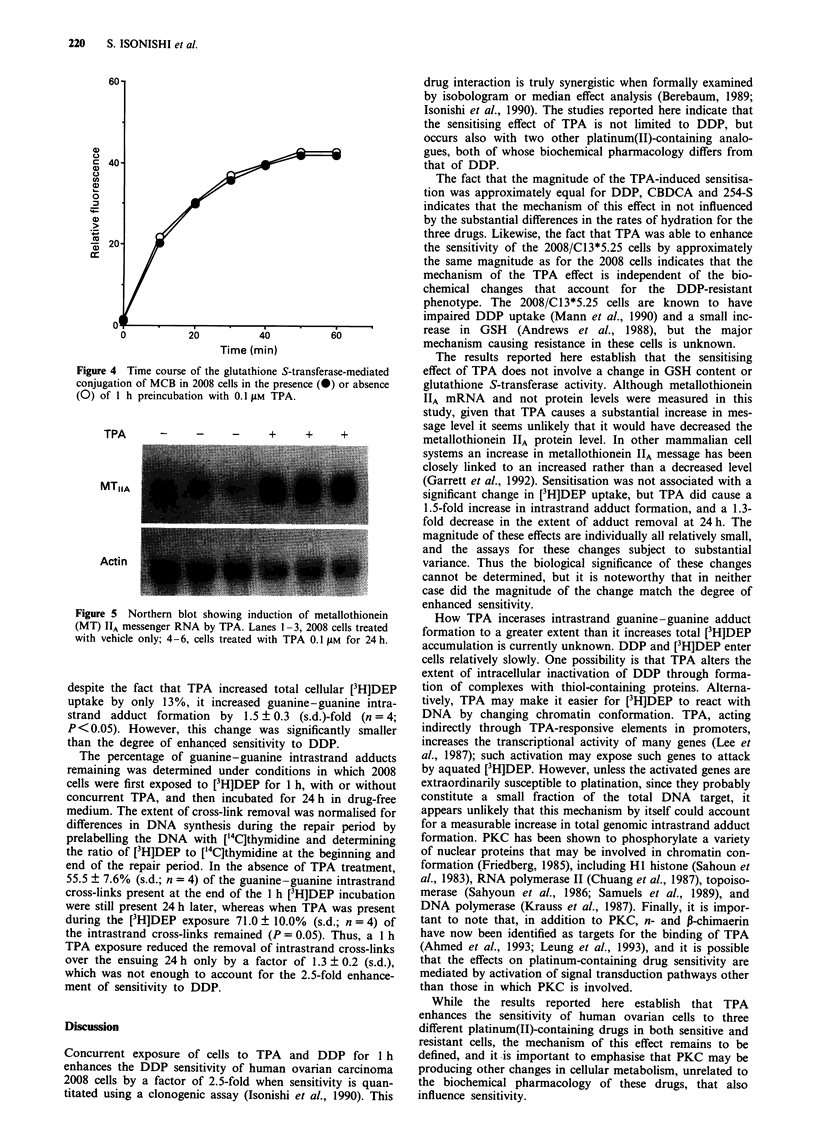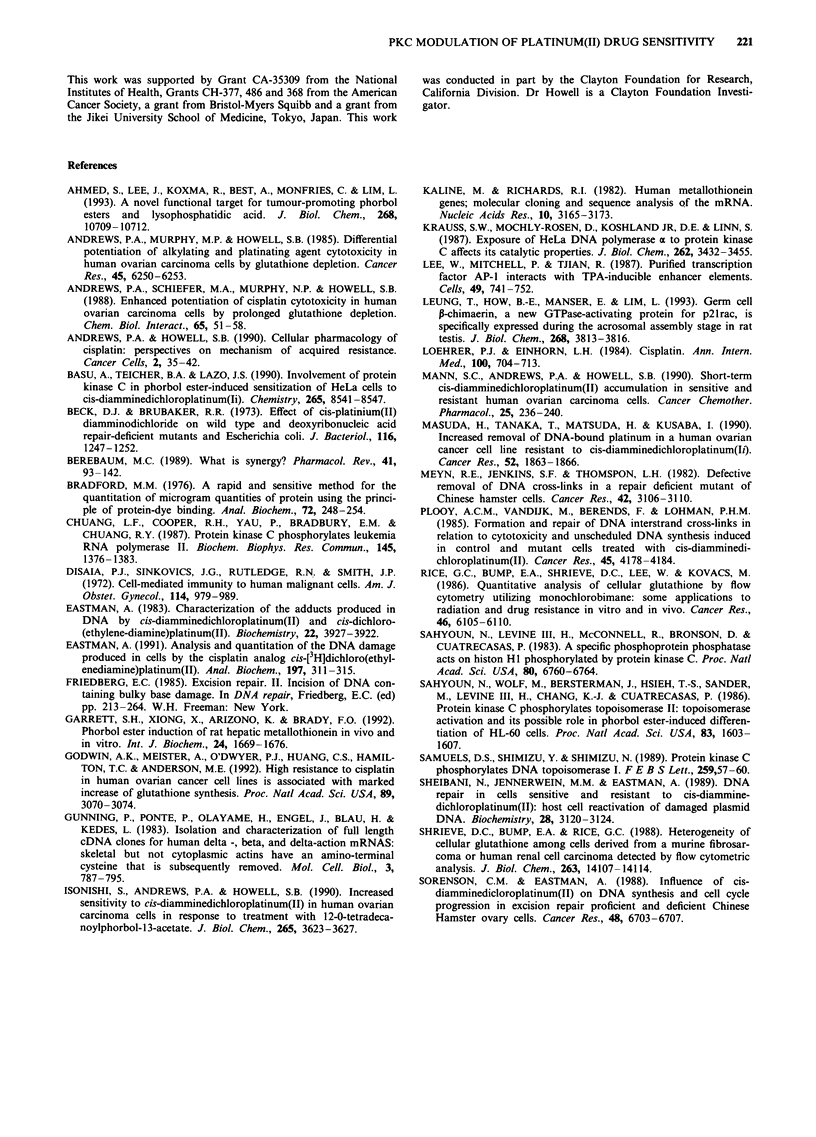# Enhancement of sensitivity to platinum(II)-containing drugs by 12-O-tetradecanoyl-phorbol-13-acetate in a human ovarian carcinoma cell line.

**DOI:** 10.1038/bjc.1994.42

**Published:** 1994-02

**Authors:** S. Isonishi, D. K. Hom, A. Eastman, S. B. Howell

**Affiliations:** Department of Obstetrics and Gynecology, Jikei University, Tokyo, Japan.

## Abstract

**Images:**


Br. J. Cancer (1994), 69, 217-221 © Macmillan Press Ltd., 1994

### Enhancement of sensitivity to platinum(II)-containing drugs by 12-O-tetradecanoyl-phorbol-13-acetate in a human ovarian carcinoma cell line

S. Isonishi[1], D.K. Hom[2], A. Eastman[3] & S.B. Howell[3]

[1]*Department of Obstetrics and Gynecology, Jikei University, Tokyo, Japan;* [2]*Department of Medicine and the Cancer Center, University of California, San Diego, La Jolla, California 92093, USA;* [3]*Department of Pharmacology, Dartmouth Medical School, Hanover, New Hampshire, USA.*

**Summary** Sensitivity to platinum-containing drugs is believed to be a function of how much drug enters the cell, the extent of DNA adduct formation and the rate at which DNA is repaired. Activation of protein kinase C by 12-O-tetradecanoyl-phorbol-13-acetate (TPA) was found to enhance the sensitivity of human ovarian carcinoma 2008 cells to cisplatin (DDP), carboplatin (CBDCA) and (glycolato-*O,O'*) diammineplatinum(II) (254-S). TPA was able to enhance the sensitivity of the DDP-resistant 2008/C13*5.25 subline to each of the three drugs to the same extent as for the 2008 cells. TPA produced no significant change in the uptake of [$^3$H]*cis*-dichloro(ethylenediamine)-platinum(II) ([$^3$H]DEP) or CBDCA. It did not alter glutathione content or glutathione-*S*-transferase activity, and induced rather than suppressed metallothionein $II_A$ mRNA levels. TPA did increase the formation of intrastrand guanine–guanine cross-links by a factor of 1.5 ± 0.3 (s.d.), and reduced the fraction of intrastrand adducts removed from DNA over the subsequent 24 h by a factor of 1.3 ± 0.2 (s.d.) ($n = 4$; $P < 0.05$), however, these effects were too small to account for the degree of TPA-induced sensitisation. These results indicate that the mechanism of TPA-induced sensitisation is not specific to any one structural form of platinum-containing drug, and that it is not readily explicable on the basis of an effect on the four major parameters currently believed to regulate DDP sensitivity.

DDP has activity against a relatively broad range of tumours (Loehrer & Einhorn, 1984), and the antineoplastic activity of this drug is generally considered to result from its reaction with DNA. The majority of the adducts formed in DNA are guanine–guanine intrastrand cross-links; less than 1% of the adducts are interstrand cross-links, and a small fraction are DNA–protein cross-links (Andrews & Howell, 1990). How these cross-links cause cell death is unknown.

Four biochemical mechanisms have been identified that can influence the sensitivity of cells to DDP (reviewed in Andrews & Howell, 1990): (1) impairment of cellular uptake; (2) elevation of glutathione-*S*-transferase (GSH) activity (Godwin *et al.*, 1992); (3) elevation of metallothioneins; and (4) variations in DNA repair. In bacteria, defects in the repair of damaged DNA are associated with hypersensitivity to DDP (Beck & Brubaker, 1973). Likewise, defects in DNA repair processes in mammalian cells render them hypersensitive to DNA-damaging agents (Meyn *et al.*, 1982). Thus, DNA repair processes are important determinants of the cytotoxic effects of DNA-damaging agents in general, and of cisplatin in particular (Plooy *et al.*, 1985; Sorenson & Eastman, 1988). Furthermore, an increase in DNA repair capability has been reported as a mechanism of resistance to genotoxic agents in DDP-resistant murine leukaemia L1210 cells (Sheibani *et al.*, 1989) and in DDP-resistant human ovarian cancer cells A2780$^{CP}$ cells (Masuda *et al.*, 1990).

We have previously reported that activation of protein kinase C (PKC) by TPA enhances the DPP sensitivity of the human ovarian carcinoma cell line 2008 (Ishonishi *et al.*, 1990), indicating that the PKC signal transduction pathway can regulate one or more of the biochemical events that determine the ability of DDP to cause cell death. This observation has been confirmed in another laboratory (Basu *et al.*, 1990). In this paper we report that activation of PKC can also sensitise cells to CBDCA and 254-S, two analogues whose biochemical pharmacology differs from that of DDP. We also report that the sensitising effect of TPA cannot be accounted for by effects on any of the four major parameters currently thought to regulate DDP sensitivity.

Correspondence: S. Isonishi, Department of Obstetrics/Gynecology, Jikei University School of Medicine, 3-25-8 Nishi-shinbashi, Minato-ku, 105 Japan.
Received 27 May 1993; and in revised form 16 September 1993.

### Materials and methods

### *Materials*

TPA was purchased from Sigma (St Louis, MO, USA). DDP and CBDCA were generous gifts from Bristol-Myers Squibb, Japan. 254-S was a generous gift from Shionogi, Japan. Monochlorobimane (MCB) was purchased from Molecular Probes (Eugene, OR, USA). A stock solution of MCB was prepared in ethanol (20 mM) and was kept at 0–5°C, protected from light. [$^{14}$C]thymidine was obtained from New England Nuclear (Boston, MA, USA). [$^3$H]DEP (specific activity ≥16.4 Ci mmol$^{-1}$, an analogue of *cis*-diamminedichloroplatinum(II) (DDP) that produces adducts at identical sites in DNA, was synthesised as previously reported (Eastman, 1983).

### *Tumour cell lines*

The human cell line 2008 was established from a patient with a serous cystadenocarcinoma of the ovary (Disaia *et al.*, 1972). The characteristics of this line and its growth conditions have been previously described (Andrews *et al.*, 1985). Sensitivity to the cytotoxic effect of the platinum compounds was determined by clonogenic assay as previously described (Isonishi *et al.*, 1990). All experiments were done using triplicate cultures of cells in logarithmic growth, and each experiment was repeated a minimum of three times.

### *[$^3$H]DEP and CBDCA uptake*

Subconfluent monolayers of 2008 cells were treated with 37°C RPMI-1640 medium containing 5 μM [$^3$H]DEP (10 μCi ml$^{-1}$) for 1 h. The medium was then aspirated, and the cells were washed rapidly four times with 4°C phosphate-buffered saline (PBS). The cells were scraped off the dishes in 200 μl of PBS and sonicated to lyse the cells; one aliquot of 25 μl was used to quantitate drug (liquid scintillation counting for total [$^3$H]DEP and another 25 μl aliquot was used for determination of protein content by the method of Bradford (1976).

### *Glutathione content and glutathione-S-transferase activity*

GSH content was measured by adjusting cells to $10^6$ ml$^{-1}$ and staining them with 25 μM MCB in complete medium at room temperature for the indicated time; relative cellular fluorescence was then immediately measured on a flow cytometer (Cytofluoro Graf IIs, Orthodiagnostic System) with excitation and emission settings of 385 and 480 nm respectively (Rice et al., 1986; Shrieve et al., 1988). Values were converted from log fluorescence to linear fluorescence intensity by application of the equation $x = 10^{[(y-20)/60]}$, where $x$ is the relative linear fluorescent intensity and $y$ is the mean log channel number. Cells that were non-viable on the basis of forward and right-angle light scatter were excluded from analysis.

The forward rate constant for the conjugation of MCB by glutathione S-transferase is given by the equation $K$ = (initial rate)/[MCB][GSH]. Since the GSH content in the uninduced and induced state turned out to be the same, and the MCB concentration was identical, the effect of TPA treatment on $K$ could be estimated from its effect on the initial slope of the conjugation curve.

### *Measurement of metallothionein messenger RNA*

Northern blots containing 10 µg of total cellular RNA were prepared by standard techniques and hybridised sequentially to probes for human metallothionein $II_A$ and β-actin (Kaline & Richards, 1982); Gunning et al., 1983), the latter of which was used to control for lane loading.

### *Extent of intrastrand cross-link formation and repair*

Subconfluent monolayers of 2008 cells were cultured with complete RPMI containing 1 nCi of [$^{14}$C]thymidine in 75 cm$^2$ culture flasks for 72 h. The medium was then changed and the cells were incubated at 37°C for 1 h with 10 µCi ml$^{-1}$ [$^3$H]DEP in the presence or absence of 0.1 µM TPA. Cells were harvested either immediately or 24 h after the treatment by trypsinisation, and washed twice with ice-cold PBS. Intrastrand guanine–guanine adducts were quantitated as previously described (Eastmen, 1983, 1991). Briefly, DNA was isolated, digested to nucleotides with deoxyribonuclease I (bovine pancreas) and P1 nuclease, and then converted to nucleosides with alkaline phosphatase. The [$^3$H]DEP-labelled guanine–guanine dinucleotide was separated and quantitated by high-performance liquid chromatography (HPLC). The extent of intrastrand cross-link removal was corrected for new DNA synthesis occurring during the repair period by expressing the amount of [$^3$H]DEP as a function of the amount of [$^{14}$C]thymidine present at each time point.

### Results

### *Modulation of sensitivity by TPA*

Figure 1 shows the structures of DDP, CDBCA and 254-S. All three are platinum(II) compounds, but each has a different leaving group. In the low-chloride environment of the cytosol, aquation displaces the two chlorides from DDP, the cyclobutane dicarboxylate from CBDCA and the glycolato group from 254-S. Thus all three drugs generate the same $Pt(NH_3)_2(H_2O)_{2++}$ reactive species, and produce the same types of adducts in DNA. Figure 2 shows a comparison of the effect of a 1 h concurrent exposure to 0.1 µM TPA on the sensitivity of human ovarian carcinoma 2008 cells to DDP, CBDCA and 254-S. TPA sensitised the cells to DDP by a factor of 2.5 ± 0.7 (s.d.), and to CBDCA and 254-S by factors of 2.8 ± 0.6 (s.d.) and 2.3 ± 0.6 (s.d.) respectively. The actual $IC_{50}$ values are presented in Table I. Sensitisation was dependent on the concentration of TPA; the effect reached a plateau at 0.1 µM, and further increases in TPA concentration did not cause any further enhancement of sensitivity (data not shown). The fact that TPA was able to sensitise cells to all three agents, and by approximately equivalent degrees, indicates that the effect was not specific to any one type of leaving group.

**Figure 1** The structures of DDP, CBDCA and 254-S

Figure 3 shows a comparison of the ability of TPA to sensitise DDP-resistant 2008/C13*5.25 cells to DDP CBDCA and 254-S. At the time of this testing, 2008/C13*5.25 cells were 4.2 ± 0.8 (s.d.)-fold resistant to DDP, 2.1 ± 0.2 (s.d.)-fold cross-resistant to CBDCA and 6.3 ± 1.5 (s.d.)-fold cross-resistant to 254-S. TPA sensitised the 2008/C13*5.25 cells by a factor of 2.7 ± 0.5 (s.d.) for DDP, 3.2 ± 0.4 (s.d.) for CBDCA and 2.5 ± 0.6 (s.d.) for 254-S (Table I). All these differences were statistically significant ($P < 0.05$). Thus, TPA was as effective at sensitising the resistant cells as it was at sensitising the sensitive 2008 parental cells, and therefore the mechanism must be independent of those factors that render the 2008/C13*5.25 cells resistant.

### *Effect of TPA on cellular uptake of [$^3$H]DEP*

The human ovarian carcinoma 2008 cells were incubated with 5 µM [$^3$H]DEP in the presence or absence of 0.1 µM TPA (or an appropriate dilution of acetone alone as a vehicle control). At 60 min the TPA-treated cells contained 113 ± 17% (s.d.) of that in the controls. Thus, there was no discernible effect of TPA on [$^3$H]DEP at uptake. We have previously shown that such an exposure to TPA by itself does not alter either the cloning efficiency or growth rate of the 2008 cells (Isonishi et al., 1990).

### *Effect of TPA on cellular GSH content and glutathione-S-transferase activity*

MCB reacts quantitatively with GSH via glutathione S-transferase to form a fluorescent product readily quantitated by flow cytometry (Rice et al., 1986; Shrieve et al., 1988). The human ovarian 2008 cells were stained with MCB for various periods of time and relative fluorescence was determined immediately by flow cytometry. Figure 4 shows a representative experiment indicating that maximum staining was obtained by 50 min, and this staining time was used for all

**Figure 2** Dose–response curves for human ovarian carcinoma 2008 cells exposed for 1 h to either DDP, CBDCA or 254-S alone (○) or concurrently to the platinum-containing drug and 0.1 μM TPA (●). Each point represents the mean of three experiments each performed with triplicate cultures. Vertical bars, s.d. Data for DDP from Isonishi *et al.* (1990) are shown for comparison.

**Figure 3** Dose–response curves for human ovarian carcinoma 2008/C13*5.25 cells exposed for 1 h to either DDP, CBDCA or 254-S alone (○) or concurrently to the platinum-containing drugs and 0.1 μM TPA (●). Each point represents the mean of three experiments each performed with triplicate cultures. Vertical bars, s.d.

**Table I** $IC_{50}$ values for DDP, CBDCA and 254-S

| *Cell type* | *TPA exposure*[a] | *$IC_{50}$ (μM) (mean ± s.d.)* *DDP* | *CBDCA* | *254-S* |
|---|---|---|---|---|
| 2008 | – | 3.1 ± 0.6 | 22.3 ± 0.1 | 8.5 ± 2.7 |
| | + | 1.2 ± 0.4 | 7.9 ± 2.4 | 3.7 ± 0.6 |
| 2008/C13*5.25 | – | 13.1 ± 2.7 | 46.2 ± 5.2 | 53.3 ± 12.6 |
| | + | 4.9 ± 1.4 | 14.6 ± 3.2 | 21.5 ± 3.2 |

[a]TPA, 0.1 μM

subsequent experiments. The GSH content of cells treated with 0.1 μM TPA for 1 h was 98.1 ± 24.7% (s.d.; $n = 3$) of that in the untreated cells. Thus, TPA treatment did not alter GSH content significantly.

Since there was no difference in GSH content, the initial rate of reaction between GSH and MCB can be used to estimate the rate constant for the glutathione *S*-transferase-mediated reaction of MCB with GSH, assuming that the MCB has equal access to the GSH in the presence and absence of TPA. The ratio of glutathione *S*-transferase activity in 2008 cells treated with or without TPA for 1 h was 1.02 ± 0.21 (s.d.; $n = 3$). Thus, TPA treatment did not produce a significant change in glutathione *S*-transferase when activity was assayed in this manner.

*Effect of TPA on cellular metallothionein mRNA*

RNA was harvested from 2008 cells 24 h after a 1 h exposure to 0.1 μM TPA, and Northern blots were probed for the level of metallothionein $II_A$ message, and subsequently with a probe for β-actin to confirm equivalent lane loading. Figure 5 shows that TPA increased the level of metallothionein $II_A$ in these cells, which is the opposite of what might be expected if TPA-induced sensitisation was working through reduction of metallothionein $II_A$ protein content.

*Effect of TPA on [$^3$H]DEP intrastrand adduct formation and repair*

Intrastrand DNA cross-link formation can be quantitated using [$^3$H]DEP as reported by Eastman (1983, 1991). This technique permits specific quantitation of the most abundant adduct, the guanine–guanine intrastrand cross-link. The 2008 cells were incubated with or without 0.1 μM TPA for 1 h concurrent with a 1 h incubation with 5 μM [$^3$H]DEP, and the extent of intrastrand cross-link formation was determined. At the end of a 1 h incubation with [$^3$H]DEP, untreated 2008 cells contained 48.6 ± 0.4 (s.d.; $n = 4$) d.p.m. per nmol of DNA phosphate, whereas 2008 cells exposed to 0.1 μM TPA for 1 h concurrently with the [$^3$H]DEP contained 67.9 ± 6.8 (s.d.; $n = 4$) d.p.m. per nmol phosphate. Thus,

**Figure 4** Time course of the glutathione *S*-transferase-mediated conjugation of MCB in 2008 cells in the presence (●) or absence (○) of 1 h preincubation with 0.1 μM TPA.

**Figure 5** Northern blot showing induction of metallothionein (MT) II$_A$ messenger RNA by TPA. Lanes 1–3, 2008 cells treated with vehicle only; 4–6, cells treated with TPA 0.1 μM for 24 h.

despite the fact that TPA increased total cellular [$^3$H]DEP uptake by only 13%, it increased guanine–guanine intrastrand adduct formation by 1.5 ± 0.3 (s.d.)-fold ($n = 4$; $P < 0.05$). However, this change was significantly smaller than the degree of enhanced sensitivity to DDP.

The percentage of guanine–guanine intrastrand adducts remaining was determined under conditions in which 2008 cells were first exposed to [$^3$H]DEP for 1 h, with or without concurrent TPA, and then incubated for 24 h in drug-free medium. The extent of cross-link removal was normalised for differences in DNA synthesis during the repair period by prelabelling the DNA with [$^{14}$C]thymidine and determining the ratio of [$^3$H]DEP to [$^{14}$C]thymidine at the beginning and end of the repair period. In the absence of TPA treatment, 55.5 ± 7.6% (s.d.; $n = 4$) of the guanine–guanine intrastrand cross-links present at the end of the 1 h [$^3$H]DEP incubation were still present 24 h later, whereas when TPA was present during the [$^3$H]DEP exposure 71.0 ± 10.0% (s.d.; $n = 4$) of the intrastrand cross-links remained ($P = 0.05$). Thus, a 1 h TPA exposure reduced the removal of intrastrand cross-links over the ensuing 24 h only by a factor of 1.3 ± 0.2 (s.d.), which was not enough to account for the 2.5-fold enhancement of sensitivity to DDP.

### Discussion

Concurrent exposure of cells to TPA and DDP for 1 h enhances the DDP sensitivity of human ovarian carcinoma 2008 cells by a factor of 2.5-fold when sensitivity is quantitated using a clonogenic assay (Isonishi *et al.*, 1990). This drug interaction is truly synergistic when formally examined by isobologram or median effect analysis (Berebaum, 1989; Isonishi *et al.*, 1990). The studies reported here indicate that the sensitising effect of TPA is not limited to DDP, but occurs also with two other platinum(II)-containing analogues, both of whose biochemical pharmacology differs from that of DDP.

The fact that the magnitude of the TPA-induced sensitisation was approximately equal for DDP, CBDCA and 254-S indicates that the mechanism of this effect in not influenced by the substantial differences in the rates of hydration for the three drugs. Likewise, the fact that TPA was able to enhance the sensitivity of the 2008/C13*5.25 cells by approximately the same magnitude as for the 2008 cells indicates that the mechanism of the TPA effect is independent of the biochemical changes that account for the DDP-resistant phenotype. The 2008/C13*5.25 cells are known to have impaired DDP uptake (Mann *et al.*, 1990) and a small increase in GSH (Andrews *et al.*, 1988), but the major mechanism causing resistance in these cells is unknown.

The results reported here establish that the sensitising effect of TPA does not involve a change in GSH content or glutathione *S*-transferase activity. Although metallothionein II$_A$ mRNA and not protein levels were measured in this study, given that TPA causes a substantial increase in message level it seems unlikely that it would have decreased the metallothionein II$_A$ protein level. In other mammalian cell systems an increase in metallothionein II$_A$ message has been closely linked to an increased rather than a decreased level (Garrett *et al.*, 1992). Sensitisation was not associated with a significant change in [$^3$H]DEP uptake, but TPA did cause a 1.5-fold increase in intrastrand adduct formation, and a 1.3-fold decrease in the extent of adduct removal at 24 h. The magnitude of these effects are individually all relatively small, and the assays for these changes subject to substantial variance. Thus the biological significance of these changes cannot be determined, but it is noteworthy that in neither case did the magnitude of the change match the degree of enhanced sensitivity.

How TPA incerases intrastrand guanine–guanine adduct formation to a greater extent than it increases total [$^3$H]DEP accumulation is currently unknown. DDP and [$^3$H]DEP enter cells relatively slowly. One possibility is that TPA alters the extent of intracellular inactivation of DDP through formation of complexes with thiol-containing proteins. Alternatively, TPA may make it easier for [$^3$H]DEP to react with DNA by changing chromatin conformation. TPA, acting indirectly through TPA-responsive elements in promoters, increases the transcriptional activity of many genes (Lee *et al.*, 1987); such activation may expose such genes to attack by aquated [$^3$H]DEP. However, unless the activated genes are extraordinarily susceptible to platination, since they probably constitute a small fraction of the total DNA target, it appears unlikely that this mechanism by itself could account for a measurable increase in total genomic intrastrand adduct formation. PKC has been shown to phosphorylate a variety of nuclear proteins that may be involved in chromatin conformation (Friedberg, 1985), including H1 histone (Sahoun *et al.*, 1983), RNA polymerase II (Chuang *et al.*, 1987), topoisomerase (Sahyoun *et al.*, 1986; Samuels *et al.*, 1989), and DNA polymerase (Krauss *et al.*, 1987). Finally, it is important to note that, in addition to PKC, *n*- and *β*-chimaerin have now been identified as targets for the binding of TPA (Ahmed *et al.*, 1993; Leung *et al.*, 1993), and it is possible that the effects on platinum-containing drug sensitivity are mediated by activation of signal transduction pathways other than those in which PKC is involved.

While the results reported here establish that TPA enhances the sensitivity of human ovarian cells to three different platinum(II)-containing drugs in both sensitive and resistant cells, the mechanism of this effect remains to be defined, and it is important to emphasise that PKC may be producing other changes in cellular metabolism, unrelated to the biochemical pharmacology of these drugs, that also influence sensitivity.

This work was supported by Grant CA-35309 from the National Institutes of Health, Grants CH-377, 486 and 368 from the American Cancer Society, a grant from Bristol-Myers Squibb and a grant from the Jikei University School of Medicine, Tokyo, Japan. This work was conducted in part by the Clayton Foundation for Research, California Division. Dr Howell is a Clayton Foundation Investigator.

### References

AHMED, S., LEE, J., KOXMA, R., BEST, A., MONFRIES, C. & LIM, L. (1993). A novel functional target for tumour-promoting phorbol esters and lysophosphatidic acid. *J. Biol. Chem.*, **268**, 10709–10712.

ANDREWS, P.A., MURPHY, M.P. & HOWELL, S.B. (1985). Differential potentiation of alkylating and platinating agent cytotoxicity in human ovarian carcinoma cells by glutathione depletion. *Cancer Res.*, **45**, 6250–6253.

ANDREWS, P.A., SCHIEFER, M.A., MURPHY, N.P. & HOWELL, S.B. (1988). Enhanced potentiation of cisplatin cytotoxicity in human ovarian carcinoma cells by prolonged glutathione depletion. *Chem. Biol. Interact.*, **65**, 51–58.

ANDREWS, P.A. & HOWELL, S.B. (1990). Cellular pharmacology of cisplatin: perspectives on mechanism of acquired resistance. *Cancer Cells*, **2**, 35–42.

BASU, A., TEICHER, B.A. & LAZO, J.S. (1990). Involvement of protein kinase C in phorbol ester-induced sensitization of HeLa cells to cis-diamminedichloroplatinum(Ii). *Chemistry*, **265**, 8541–8547.

BECK, D.J. & BRUBAKER, R.R. (1973). Effect of cis-platinium(II) diamminodichloride on wild type and deoxyribonucleic acid repair-deficient mutants and Escherichia coli. *J. Bacteriol.*, **116**, 1247–1252.

BEREBAUM, M.C. (1989). What is synergy? *Pharmacol. Rev.*, **41**, 93–142.

BRADFORD, M.M. (1976). A rapid and sensitive method for the quantitation of microgram quantities of protein using the principle of protein-dye binding. *Anal. Biochem.*, **72**, 248–254.

CHUANG, L.F., COOPER, R.H., YAU, P., BRADBURY, E.M. & CHUANG, R.Y. (1987). Protein kinase C phosphorylates leukemia RNA polymerase II. *Biochem. Biophys. Res. Commun.*, **145**, 1376–1383.

DISAIA, P.J., SINKOVICS, J.G., RUTLEDGE, R.N. & SMITH, J.P. (1972). Cell-mediated immunity to human malignant cells. *Am. J. Obstet. Gynecol.*, **114**, 979–989.

EASTMAN, A. (1983). Characterization of the adducts produced in DNA by *cis*-diamminedichloroplatinum(II) and *cis*-dichloro-(ethylene-diamine)platinum(II). *Biochemistry*, **22**, 3927–3922.

EASTMAN, A. (1991). Analysis and quantitation of the DNA damage produced in cells by the cisplatin analog *cis*-[$^3$H]dichloro(ethylenediamine)platinum(II). *Anal. Biochem.*, **197**, 311–315.

FRIEDBERG, E.C. (1985). Excision repair. II. Incision of DNA containing bulky base damage. In *DNA repair*, Friedberg, E.C. (ed) pp. 213–264. W.H. Freeman: New York.

GARRETT, S.H., XIONG, X., ARIZONO, K. & BRADY, F.O. (1992). Phorbol ester induction of rat hepatic metallothionein in vivo and in vitro. *Int. J. Biochem.*, **24**, 1669–1676.

GODWIN, A.K., MEISTER, A., O'DWYER, P.J., HUANG, C.S., HAMILTON, T.C. & ANDERSON, M.E. (1992). High resistance to cisplatin in human ovarian cancer cell lines is associated with marked increase of glutathione synthesis. *Proc. Natl Acad. Sci. USA*, **89**, 3070–3074.

GUNNING, P., PONTE, P., OLAYAME, H., ENGEL, J., BLAU, H. & KEDES, L. (1983). Isolation and characterization of full length cDNA clones for human delta -, beta, and delta-action mRNAS: skeletal but not cytoplasmic actins have an amino-terminal cysteine that is subsequently removed. *Mol. Cell. Biol.*, **3**, 787–795.

ISONISHI, S., ANDREWS, P.A. & HOWELL, S.B. (1990). Increased sensitivity to *cis*-diamminedichloroplatinum(II) in human ovarian carcinoma cells in response to treatment with 12-0-tetradecanoylphorbol-13-acetate. *J. Biol. Chem.*, **265**, 3623–3627.

KALINE, M. & RICHARDS, R.I. (1982). Human metallothionein genes; molecular cloning and sequence analysis of the mRNA. *Nucleic Acids Res.*, **10**, 3165–3173.

KRAUSS, S.W., MOCHLY-ROSEN, D., KOSHLAND JR, D.E. & LINN, S. (1987). Exposure of HeLa DNA polymerase α to protein kinase C affects its catalytic properties. *J. Biol. Chem.*, **262**, 3432–3455.

LEE, W., MITCHELL, P. & TJIAN, R. (1987). Purified transcription factor AP-1 interacts with TPA-inducible enhancer elements. *Cells*, **49**, 741–752.

LEUNG, T., HOW, B.-E., MANSER, E. & LIM, L. (1993). Germ cell β-chimaerin, a new GTPase-activating protein for p21rac, is specifically expressed during the acrosomal assembly stage in rat testis. *J. Biol. Chem.*, **268**, 3813–3816.

LOEHRER, P.J. & EINHORN, L.H. (1984). Cisplatin. *Ann. Intern. Med.*, **100**, 704–713.

MANN, S.C., ANDREWS, P.A. & HOWELL, S.B. (1990). Short-term cis-diamminedichloroplatinum(II) accumulation in sensitive and resistant human ovarian carcinoma cells. *Cancer Chemother. Pharmacol.*, **25**, 236–240.

MASUDA, H., TANAKA, T., MATSUDA, H. & KUSABA, I. (1990). Increased removal of DNA-bound platinum in a human ovarian cancer cell line resistant to cis-diamminedichloroplatinum(I*i*). *Cancer Res.*, **52**, 1863–1866.

MEYN, R.E., JENKINS, S.F. & THOMSPON, L.H. (1982). Defective removal of DNA cross-links in a repair deficient mutant of Chinese hamster cells. *Cancer Res.*, **42**, 3106–3110.

PLOOY, A.C.M., VANDIJK, M., BERENDS, F. & LOHMAN, P.H.M. (1985). Formation and repair of DNA interstrand cross-links in relation to cytotoxicity and unscheduled DNA synthesis induced in control and mutant cells treated with cis-diamminedichloroplatinum(II). *Cancer Res.*, **45**, 4178–4184.

RICE, G.C., BUMP, E.A., SHRIEVE, D.C., LEE, W. & KOVACS, M. (1986). Quantitative analysis of cellular glutathione by flow cytometry utilizing monochlorobimane: some applications to radiation and drug resistance in vitro and in vivo. *Cancer Res.*, **46**, 6105–6110.

SAHYOUN, N., LEVINE III, H., McCONNELL, R., BRONSON, D. & CUATRECASAS, P. (1983). A specific phosphoprotein phosphatase acts on histon H1 phosphorylated by protein kinase C. *Proc. Natl Acad. Sci. USA*, **80**, 6760–6764.

SAHYOUN, N., WOLF, M., BERSTERMAN, J., HSIEH, T.-S., SANDER, M., LEVINE III, H., CHANG, K.-J. & CUATRECASAS, P. (1986). Protein kinase C phosphorylates topoisomerase II: topoisomerase activation and its possible role in phorbol ester-induced differentiation of HL-60 cells. *Proc. Natl Acad. Sci. USA*, **83**, 1603–1607.

SAMUELS, D.S., SHIMIZU, Y. & SHIMIZU, N. (1989). Protein kinase C phosphorylates DNA topoisomerase I. *F E B S Lett.*, **259**,57–60.

SHEIBANI, N., JENNERWEIN, M.M. & EASTMAN, A. (1989). DNA repair in cells sensitive and resistant to cis-diamminedichloroplatinum(II): host cell reactivation of damaged plasmid DNA. *Biochemistry*, **28**, 3120–3124.

SHRIEVE, D.C., BUMP, E.A. & RICE, G.C. (1988). Heterogeneity of cellular glutathione among cells derived from a murine fibrosarcoma or human renal cell carcinoma detected by flow cytometric analysis. *J. Biol. Chem.*, **263**, 14107–14114.

SORENSON, C.M. & EASTMAN, A. (1988). Influence of cis-diamminedicloroplatinum(II) on DNA synthesis and cell cycle progression in excision repair proficient and deficient Chinese Hamster ovary cells. *Cancer Res.*, **48**, 6703–6707.